# Evaluation of Whole Blood Viscosity in Patients with Aortic Sclerosis

**Published:** 2017-01

**Authors:** Pınar Türker Duyuler, Serkan Duyuler, Mehmet İleri, Mevlüt Demir, Abdullah Kadir Dolu, Funda Başyiğit

**Affiliations:** 1 *Department of Cardiology, Ankara Numune Training and Research Hospital, Ankara, Turkey.*; 2 *Department of Cardiology, Acıbadem Ankara Hospital, Ankara, Turkey.*

**Keywords:** *Aortic valve sclerosis*, *Blood viscosity*, *Atherosclerosis*

## Abstract

**Background: **Blood viscosity and aortic sclerosis (AS) are strong predictors of cardiovascular events. The effects of blood viscosity on AS have not been studied adequately. We aimed to investigate the potential connection between whole blood viscosity (WBV) and AS.

**Methods:** AS was detected by transthoracic echocardiography. The estimation of WBV was carried out at both high shear rate (HSR) (208/s) and low shear rate (LSR) (0.5/s) by previously validated formulae using hematocrit (HcT) and total protein (TP) in g/L. WBV at HSR (208/s) is: (0.12 × HcT) + 0.17 (TP - 2.07) and WBV at LSR (0.5/s) is: (1.89 × HcT) + 3.76 (TP - 78.42). Comparisons of WBV at both HSR and LSR were made between patients with and without AS.

**Results: **We included 94 patients with AS (male = 30.9%, mean age = 67.5 y) and 97 control subjects without AS (male =26.6%, mean age = 69.1 y). Almost all of the clinical, echocardiographic, and biochemical characteristics were similar, but TP values were significantly higher in the AS group than in the control group (72.9 ± 5 g/L vs. 75.8 ± 6.1 g/L; p value < 0.001). Hemoglobin and HcT levels were similar (p value = 0.604 and p value = 0.431, respectively). In the AS group, WBV at LSR and HSR was higher than that in the control group (p value = 0.001 for both LSR and HSR). In multiple stepwise logistic regression analysis, WBV was an independent predictor of AS (p value < 0.001).

**Conclusion** **:** We found higher WBV in patients with AS than in patients without AS at both LSR (0.5/s) and HSR (208/s). WBV at both LSR and HSR was independently associated with AS.

## Introduction

Blood viscosity is the internal dynamic resistance of blood to flow and determines the frictional force applied against the vessel walls. The determinants of blood viscosity are plasma viscosity, hematocrit (HcT), and aggregation and deformation abilities of erythrocytes, making blood a non-Newtonian fluid.^1^ Whole blood viscosity (WBV) may be predicted by previously validated formulas using HcT and plasma protein.^2^ Major risk factors for atherosclerosis such as high blood cholesterol, hypertension, aging, diabetes, metabolic syndrome, and obesity are also related with increased blood viscosity.^3^ Studies suggest that increased blood viscosity is at least as strong a predictor of cardiovascular events as diastolic blood pressure and low-density lipoprotein (LDL) cholesterol.^4^ Aortic valve sclerosis is the thickening of the aortic valve without a significant obstruction of the outflow, which may or may not accompany valvular calcification. It is not an infrequent finding with aging during cardiac imaging, and leaflet restriction is not hemodynamically significant,^5^ which is expressed by a maximal transvalvular velocity < 2–2.5 m/s. Aortic valve stenosis is a precursor of aortic stenosis (AS) and an important predictor of coronary artery disease.^6^ Although coronary artery disease and AS share histopathological and etiological similarities, medical therapies such as statins have no effect on stopping or slowing AS,^5^ which necessitates the identification of other potential risk factors and, thus, new therapeutic options. On the other hand, the effects of blood viscosity on AS have not been studied adequately. In this context, we aimed to investigate the potential relationship between WBV and AS.

## Methods

We evaluated 94 patients with AS and 97 age- and gender-matched control subjects without AS selected from among individuals referred to the Echocardiography Laboratory of Ankara Numune Training and Research Hospital. Patients with AS, moderate-to-severe valvular disease, bicuspid aortic valve, history of rheumatic heart disease, left ventricular systolic dysfunction, symptomatic coronary artery disease, history of chronic kidney disease, paraproteinemia, acute blood loss or surgery, and history of hematological disease and anemia according to the patients’ self-report and medical records were excluded from the study. Hypertension was defined as blood pressure > 140/90 mmHg or use of antihypertensive drugs. Diabetes mellitus was defined as fasting blood glucose > 125 mg/dL or use of antidiabetic treatment. Hypercholesterolemia was defined as total cholesterol > 200 mg/dL or use of antilipidemic treatment. Laboratory tests for fasting blood glucose, lipid profile, creatinine levels, complete blood count, and total protein (TP) were performed with standard methods using commercially available laboratory kits.

AS was detected by using a commercially available echocardiography device (Vivid 7 pro, GE Vingmed, Horten, Norway) with a 2.5–3.5 MHz transducer. AS was defined as focal areas of increased echogenity and thickening of the aortic trileaflet valve without valve motion restriction and left ventricular outflow tract obstruction (aortic jet velocity < 2.5 m/s with continuous wave Doppler ultrasound). The modified Simpson method was used to estimate the left ventricular ejection fraction. The diameter of the ascending aortic aorta and the presence of mitral annular calcification were also recorded. These measurements and evaluations were performed by 2 experienced cardiologists in accordance with the current recommendations of practice guidelines.^7^^, ^^8^

The estimation of WBV was carried out in both high shear rate (HSR = 208/s) and low shear rate (LSR = 0.5/s) via previously validated formulas,^2^^, ^^9^^, ^^10^ which utilize HcT and total plasma protein concentration. For HSR, the WBV (208/s) formula is as follows: (0.12 × HcT) + 0.17 (TP – 2.07) and for LSR, WBV (0.5/ s) is: (1.89 × HcT) + 3.76 (TP – 78.42), where HcT is hematocrit in %, TP is total protein concentration in g/L, and WBV is whole blood viscosity in centipoise (cP).

The statistical analyses were performed with IBM SPSS for Windows, version 22.0. The continuous variables are expressed as means ± SDs or medians (minimum – maximum), where appropriate, and the categorical variables are expressed in percentages. The parametric test assumptions (normality and homogeneity of the variances) were tested before the groups were compared in terms of the continuous variables. The Shapiro–Wilk test was used when the continuous variables were not normally distributed. The homogeneity of the variances was tested using the Levine test. The continuous variables were tested with the *t*-test or the Mann– Whitney *U*-test, where appropriate. The categorical variables were evaluated with the χ² test or the Fischer exact test. Factors affecting AS were determined with multiple stepwise logistic regression analysis. A p value < 0.05 was considered statistically significant.

## Results

The baseline clinical, echocardiographic, and biochemical characteristics of the study groups are summarized in Table 1. Almost all the clinical, echocardiographic, and biochemical characteristics were similar in both groups. Aortic velocity was significantly higher in the patients with AS than in the control patients, as was expected (1.3 [IQR: 1.1 – 2] m/s vs. 1.9 [IQR: 1.8 – 2.3] m/s; p value < 0.001). TP values were significantly higher in the AS group than in the control group (72.9 ± 5 g/L vs. 75.8 ± 6.1 g/L; p value < 0.001). On the other hand, hemoglobin and HcT levels were similar in both groups (p value = 0.604 and p value = 0.431, respectively). High-density lipoprotein (HDL) cholesterol levels were significantly lower in the AS group than in the control group (45.9 ± 12.3 mg/dL vs. 49.8 ± 12.8 mg/dl; p value = 0.034), and LDL cholesterol levels tended to be higher in the AS group, but the difference was statistically insignificant (45.9 ± 12.3 mg/dL vs. 49.8±12.8 mg/dL; p value = 0.034). LDL cholesterol levels tended to be higher in the AS group, but the difference was statistically insignificant (147.2 ± 45.1 vs. 136.4 ± 38.8; p value = 0.079). WBV in LSR and HSR was higher in the AS group than in the control group (p value = 0.001 for both LSR and HSR) (Table 2).

In the multiple stepwise logistic regression analysis, AS was taken as a dependent variable. LDL, HDL, platelet count, ejection fraction, presence of mitral annular calcification, and WBV at LSR were included in Model 1 and LDL, HDL, platelet count, ejection fraction, presence of mitral annular calcification, and WBV at HSR were included in Model 2. In both models, WBV was an independent predictor of AS (p value < 0.001) (Table 3).

**Table 1:** Clinical, biochemical, and anthropometric characteristics of the 2 groups with and without aortic sclerosis*

	Aortic Sclerosis (-) (n=97)	Aortic Sclerosis (+) (n=94)	P value
Age (y)	67.5±8.0	67.1±9.2	0.774
Sex (male)	30 (30.9)	25 (26.6)	0.509
Body mass index (kg/m^2)^	25.20±1.4	25.47±1.71	0.168
Smoking	39 (40.2)	35 (37.2)	0.785
Diabetes mellitus	32 (33.0)	23 (24.7)	0.210
Hyperlipidemia	40 (41.2)	35 (37.2)	0.571
Hypertension	73 (75.3)	75 (79.8)	0.565
Coronary artery disease	35 (36.5)	35 (37.2)	1.000
LVEF (%)	63 (50 – 75)	65 (50 – 78)	0.110
Aortic jet velocity (m/s)	1.3 (1.1 – 2)	1.9 (1.8 – 2.3)	< 0.001
MAC	5 (5.2)	21 (22.6)	0.001
Urea (mg/dL)	33 (16 – 69)	36 (17 – 90)	0.309
Serum creatinine (mg/dL)	0.83 (0.5 – 1.6)	0.83 (0.5 – 1.7)	0.406
White blood cell count (10^3^/uL)	7.4±1.6	7.5±1.9	0.711
Platelet (10^3^/uL)	245.7±64.3	261.9±74.5	0.109
Hemoglobin (g/dL)	13.6±1.3	13.7±1.3	0.604
Hematocrit (%)	40.8±3.7	41.2±3.8	0.431
Total protein (g/L)	72.9±5.0	75.8±6.1	< 0.001
Fasting serum glucose (mg/dL)	102 (73 – 348)	106.5 (75 – 275)	0.570
Total cholesterol (mg/dL)	216.7±44.5	219.8±55.6	0.665
Triglycerides (mg/dL)	130 (32 – 552)	139 (55 – 397)	0.450
LDL cholesterol (mg/dL)	136.4±38.8	147.2±45.1	0.079
HDL cholesterol (mg/dL)	49.8±12.8	45.9±12.3	0.034
Statins	36 (37.1)	34 (36.2)	0.892
Beta-blocker	37 (38.1)	36 (38.3)	0.983
ACE inhibitor /ARB	69 (71.1)	66 (70.2)	0.889
Acetyl salicylic acid	36 (37.1)	39 (41.5)	0.536

LVEF, Left ventricular ejection fraction; MAC, Mitral annular calcification; LDL, Low-density lipoprotein; HDL, High-density lipoprotein; ARB, Angiotensin receptor blockers

* Data are presented as mean±SD, median (interquartile range), or n (%).

**Table 2:** Whole blood viscosity (WBV) of the patients at high shear rate (HSR) and low shear rate (LSR)*

	Aortic Sclerosis (-) (n=97)	Aortic Sclerosis (+) (n=94)	P value
WBV at HSR, 208 s^-1^	56.4±20.9	68.2±25.9	0.001
WBV at LSR, 0.5 s^-1^	16.9±1.0	17.5±1.2	0.001

* Data are presented as mean±SD.

**Table 3:** Stepwise logistic regression models of factors independently associated with aortic sclerosis

	OR	95% CI	P value
Model 1			
HDL	0.949	0.921 – 0.978	0.001
LDL	1.009	1.001 – 1.018	0.028
LSR	1.028	1.013 – 1.043	< 0.001
LVEF	1.085	1.001 – 1.177	0.048
MAC	8.560	2.679 – 27.351	< 0.001
			
Model 2			
HDL	0.950	0.922 – 0.978	0.001
LDL	1.009	1.001 – 1.018	0.030
HSR	1.732	1.279 – 2.344	< 0.001
LVEF	1.084	1.000 – 1.175	0.050
MAC	8.623	2.697 – 27.572	< 0.001

HDL, High-density lipoprotein; LDL, Low-density lipoprotein; LSR, Low shear rate; LVEF, Left ventricular ejection fraction; MAC, Mitral annular calcification; HSR, High shear rate

## Discussion

In the present study, we found that, by comparison with patients without AS, patients with AS had high WBV at both LSR (0.5/s) and HSR (208/s). WBV at both LSR and HSR was an independent determinant of AS. This result suggests that WBV may have a potential role in the development of AS. AS shows a prevalence of about 10 % and an incidence of 1.7% to 8.8%. The progression of AS to clinical AS per year is < 2%.^5^ Although the progression rate of AS is low, studies suggest that AS is closely associated with increased cardiovascular events and mortality.^11^^, ^^12^ Current medical treatments like statins do not slow or stop the progression of AS.^13^ In our study, we did not find a statistical difference between the AS and control groups apropos medical treatment. In this regard, the potential risk factors that may play a role in AS development and progression other than conventional ones should be determined with a view to improving therapeutic options. Dyslipidemia, hypertension, smoking, inheritance, obesity, diabetes, and metabolic syndrome are well-known risk factors for vascular sclerosis. However, a substantial portion of individuals with vascular sclerosis are free from classical risk factors. In this context, about 20% of myocardial infarctions are seen in the absence of these risk factors.^14^ Conventional risk factors fail to adequately depict the full picture. Elevated WBV is associated with an increased risk of acute myocardial infarction and cardiovascular death.^15^^, ^^16^ WBV may be the neglected part of this picture.

The significance of acute hyperviscosity syndromes like polycythemia vera and leukemia is well known, and immediate intervention is necessary. However, lesser elevation in viscosity in a chronic manner is not appreciated adequately. Acute hyperviscosity syndromes possess analogy with hypertensive crisis; and just like chronic hypertension, chronic hyperviscosity is associated with a shorter life expectancy. Both conditions may operate in synergy. Additionally, authors have suggested that chronic hyperviscosity be treated in the same manner as chronic hypertension.^14^ In our study, the prevalence of hypertension was not different between the groups with and without AS; nonetheless, WBV was elevated in the group with AS, which also shows the significance of hyperviscosity in the absence of hypertension.

One of the main mechanisms triggering valvular sclerosis is inflammation, which is also related with the activation of the leaflet endothelium with an increase in cell adhesion molecules and proinflammatory cytokines. Shear stress abnormalities may enhance the expression of proinflammatory genes and trigger tissue mineralization.^17^^, ^^18^ Increased viscosity is associated with increased shear stress for a given shear rate, and increased WBV may trigger valvular inflammation by increasing shear stress. In our study, the presence of mitral annular calcification in the patients was independently associated with AS. Both conditions are not simple degenerative processes and have similarities to atherosclerosis inasmuch as they share some risk factors. In a recent study, Çetin et al.^19^ demonstrated that WBV was an independent predictor of the presence of mitral annular calcification and limitation in annular motion. We also found that WBV was an independent predictor of AS apart from the presence of mitral annular calcification.

Our study has some limitations. In our study, we did not measure WBV directly by using a viscometer. Instead, we used previously validated equations for estimating WBV. Actual measurements may differ from the estimated viscosity calculated with regression-based equations. We employed single measurements of TP and HcT to calculate WBV. However, AS is a chronic process and multiple measurements at wider time intervals may be more appropriate to show the interaction.

## Conclusion

Higher whole blood viscosity levels were independently associated with aortic sclerosis at both high shear rate and low shear rate. 

## Notes:

***This paper should be cited as:*** Türker Duyuler P, Duyuler S, İleri M, Demir M, Dolu AK, Başyiğit F. Evaluation of Whole Blood Viscosity in Patients with Aortic Sclerosis. J Teh Univ Heart Ctr 2017;12(1):6-10.

## References

[1] Dikmenoğlu N, Ciftçi B, Ileri E, Güven SF, Seringeç N, Aksoy Y, Ercil D (2006). Erythrocyte deformability, plasma viscosity and oxidative status in patients with severe obstructive sleep apnea syndrome. Sleep Med.

[2] de Simone G, Devereux RB, Chien S, Alderman MH, Atlas SA, Laragh JH (1990). Relation of blood viscosity to demographic and physiologic variables and to cardiovascular risk factors in apparently normal adults. Circulation.

[3] Sloop GD, Garber DW (1997). The effects of low-density lipoprotein and high-density lipoprotein on blood viscosity correlate with their association with risk of atherosclerosis in humans. Clin Sci (Lond).

[4] Lowe GD, Lee AJ, Rumley A, Price JF, Fowkes FG (1997). Blood viscosity and risk of cardiovascular events: the Edinburgh Artery Study. Br J Haematol.

[5] Coffey S, Cox B, Williams MJ (2014). The prevalence, incidence, progression, and risks of aortic valve sclerosis: a systematic review and meta-analysis. J Am Coll Cardiol.

[6] Taylor HA, Jr, Clark BL, Garrison RJ, Andrew ME, Han H, Fox ER, Arnett DK, Samdarshi T, Jones DW (2005). Relation of aortic valve sclerosis to risk of coronary heart disease in African-Americans. Am J Cardiol.

[7] Baumgartner H, Hung J, Bermejo J, Chambers JB, Evangelista A, Griffin BP, Iung B, Otto CM, Pellikka PA, Quiñones M, American Society of Echocardiography; European Association of Echocardiography (2009). Echocardiographic assessment of valve stenosis: EAE/ASE recommendations for clinical practice. J Am Soc Echocardiogr.

[8] Lang RM, Bierig M, Devereux RB, Flachskampf FA, Foster E, Pellikka PA, Picard MH, Roman MJ, Seward J, Shanewise J, Solomon S, Spencer KT, St John Sutton M, Stewart W, American Society of Echocardiography's Nomenclature and Standards Committee, Task Force on Chamber Quantification, American College of Cardiology Echocardiography Committee, American Heart Association, European Association of Echocardiography, European Society of Cardiology, American Society of Echocardiography Nomenclature and Standards Committee, Task Force on Chamber Quantification, American College of Cardiology Echocardiography Committee, American Heart Association, European Association of Echocardiography, European Society of Cardiology (2006). Recommendations for chamber quantification. Eur J Echocardiogr.

[9] Tamariz LJ, Young JH, Pankow JS, Yeh HC, Schmidt MI, Astor B, Brancati FL (2008). Blood viscosity and hematocrit as risk factors for type 2 diabetes mellitus: the atherosclerosis risk in communities (ARIC) study. Am J Epidemiol.

[10] Nwose EU, Richards RS (2011). Whole blood viscosity extrapolation formula: note on appropriateness of units. N Am J Med Sci.

[11] Otto CM, Lind BK, Kitzman DW, Gersh BJ, Siscovick DS (1999). Association of aortic-valve sclerosis with cardiovascular mortality and morbidity in the elderly. N Engl J Med.

[12] Owens DS, Budoff MJ, Katz R, Takasu J, Shavelle DM, Carr JJ, Heckbert SR, Otto CM, Probstfield JL, Kronmal RA, O'Brien KD (2012). Aortic valve calcium independently predicts coronary and cardiovascular events in a primary prevention population. JACC Cardiovasc Imaging.

[13] Chan KL, Teo K, Dumesnil JG, Ni A, Tam J, ASTRONOMER Investigators (2010). Effect of Lipid lowering with rosuvastatin on progression of aortic stenosis: results of the aortic stenosis progression observation: measuring effects of rosuvastatin (ASTRONOMER) trial. Circulation.

[14] Sloop G, Holsworth RE Jr, Weidman JJ, St Cyr JA (2015). The role of chronic hyperviscosity in vascular disease. Ther Adv Cardiovasc Dis.

[15] Danesh J, Collins R, Peto R, Lowe GD (2000). Haematocrit, viscosity, erythrocyte sedimentation rate: meta-analyses of prospective studies of coronary heart disease. Eur Heart J.

[16] Lowe GD, Fowkes FG, Dawes J, Donnan PT, Lennie SE, Housley E (1993). Blood viscosity, fibrinogen, and activation of coagulation and leukocytes in peripheral arterial disease and the normal population in the Edinburgh Artery Study. Circulation.

[17] Balachandran K, Sucosky P, Yoganathan AP (2011). Hemodynamics and mechanobiology of aortic valve inflammation and calcification. Int J Inflam.

[18] Sucosky P, Balachandran K, Elhammali A, Jo H, Yoganathan AP (2009). Altered shear stress stimulates upregulation of endothelial VCAM-1 and ICAM-1 in a BMP-4- and TGF-beta1-dependent pathway. Arterioscler Thromb Vasc Biol.

[19] Ozcan Cetin EH, Cetin MS, Canpolat U, Kalender E, Topaloglu S, Aras D, Aydogdu S (2015). The forgotten variable of shear stress in mitral annular calcification: whole blood viscosity. Med Princ Pract.

